# Supplementary material for: Dynalign II: common secondary structure prediction for RNA homologs with domain insertions
Source: Nucleic Acids Res. 2014 Nov 21;42(22):13939–48. doi: 10.1093/nar/gku1172 (PMC4267632; doi:10.1093/nar/gku1172)
Supplement: SUPPLEMENTARY DATA [file supp_gku1172_nar-02021-z-2014-File012.zip › manual/javadoc/comments/DrawingSourceDialogs.html]

A package that holds all Java drawing dialogs.
  
  
The drawing dialogs are in a hierarchy, with the base class handling all common dialog tasks, and the subclasses handling specific painting routines.
Subclasses also contain other methods besides painting routines that contain specialized GUI actions.
